# Group Dynamics of Zebra and Wildebeest in a Woodland Savanna: Effects of Predation Risk and Habitat Density

**DOI:** 10.1371/journal.pone.0012758

**Published:** 2010-09-20

**Authors:** Maria Thaker, Abi T. Vanak, Cailey R. Owen, Monika B. Ogden, Rob Slotow

**Affiliations:** 1 School of Biological and Conservation Sciences, University of KwaZulu-Natal, Durban, South Africa; 2 Karongwe Ecological Research Institute, Schagen, South Africa; University of California, United States of America

## Abstract

**Background:**

Group dynamics of gregarious ungulates in the grasslands of the African savanna have been well studied, but the trade-offs that affect grouping of these ungulates in woodland habitats or dense vegetation are less well understood. We examined the landscape-level distribution of groups of blue wildebeest, *Connochaetes taurinus*, and Burchell's zebra, *Equus burchelli*, in a predominantly woodland area (Karongwe Game Reserve, South Africa; KGR) to test the hypothesis that group dynamics are a function of minimizing predation risk from their primary predator, lion, *Panthera leo*.

**Methodology/Principal Findings:**

Using generalized linear models, we examined the relative importance of habitat type (differing in vegetation density), probability of encountering lion (based on utilization distribution of all individual lions in the reserve), and season in predicting group size and composition. We found that only in open scrub habitat, group size for both ungulate species increased with the probability of encountering lion. Group composition differed between the two species and was driven by habitat selection as well as predation risk. For both species, composition of groups was, however, dominated by males in open scrub habitats, irrespective of the probability of encountering lion.

**Conclusions/Significance:**

Distribution patterns of wildebeest and zebra groups at the landscape level directly support the theoretical and empirical evidence from a range of taxa predicting that grouping is favored in open habitats and when predation risk is high. Group composition reflected species-specific social, physiological and foraging constraints, as well as the importance of predation risk. Avoidance of high resource open scrub habitat by females can lead to loss of foraging opportunities, which can be particularly costly in areas such as KGR, where this resource is limited. Thus, landscape-level grouping dynamics are species specific and particular to the composition of the group, arising from a tradeoff between maximizing resource selection and minimizing predation risk.

## Introduction

Group formation is common in animals [1], but the size and composition of groups are temporally and spatially dynamic, and depend on the relative costs and benefits of grouping. Individuals in larger groups benefit from collective vigilance, cooperative defense, and dilution and confusion effects which can reduce predation risk [1]. Groups also gain benefits for resource acquisition [2] via information about environmental quality gained through social foraging [3], [4]. These benefits of grouping are weighed against the costs of sharing food [5], [6], and of increased probability of being detected by predators [7]. Often, the antipredator benefits of grouping may outweigh resource acquisition and social benefits [1]. Given these trade-offs, groups should vary in size and composition, and be non-uniform in their distribution across the landscape [8].

Environmental characteristics that affect the cost-benefit trade-off of grouping vary in space and time. Thus, in species where grouping is an antipredator strategy, group size and membership should depend on key factors such as the level of predation risk, habitat conditions and season. For example, forming larger groups may be more effective against stalking predators than ambush predators, especially since stalking predators often target individual prey [9] or smaller groups [10]. Furthermore, larger groups should be favored in open habitats to counteract the unavoidable danger of being detected by predators [7], while smaller groups can reduce their probability of being detected in forested habitats [11]. Predation risk also changes with season and breeding status [12]. For example, adult males of African ungulates are most at risk during the mating season [13], while adult females suffer highest predation risk during late gestation and immediately following parturition [14]. Therefore, vulnerability to predation risk should also depend on the composition of the group. In cercopithecoid primates, group composition is skewed towards adult males under conditions of high predation risk [15], while studies of free ranging fish show that individuals actively alter shoal composition [16] to reduce predation risk as well as food competition [17]. Thus group size and composition should not only change with environmental conditions, but the location of groups should alter to minimize predation risk for individuals in the group.

Some of the most well studied and dramatic examples of grouping are seen in gregarious ungulates in the grassland and open scrub habitats of the African savanna [18]. However, group dynamics and the trade-offs that affect grouping of African ungulates in closed habitats or dense vegetation are less well understood. Here we examine the landscape-level distribution of groups of blue wildebeest, *Connochaetes taurinus*, and Burchell's zebra, *Equus burchelli*, to test the hypothesis that group size and group composition are a function of minimizing predation risk. We conducted this study in a predominantly woodland landscape in the Karongwe Game Reserve, South Africa, where these two ungulate species occur at relatively similar densities (2.5±0.5 SD wildebeest/km^2^; 1.8±0.2 SD zebra/km^2^) and are known to form mixed-sex and -age herds. Lion, *Panthera leo*, are the primary predators of zebra and wildebeest in this multi-predator landscape [19], and kill adult males and females, as well as juveniles of these species [20]. If grouping is an antipredator strategy in this landscape, we predict that group sizes for both species will be larger in open habitats, especially when predation risk from lion is high. In our study area, adult females of both ungulate species suffer greater mortality from lion than adult males and young [20]. Therefore, we also predict that in closed habitats and areas where lion activity is low, group composition will be skewed toward females, especially during the calving season when females are most vulnerable.

## Methods

Ethics approval for handling animals, in strict accordance with good animal practice, was obtained from the Animal Ethics Committee of the University of KwaZulu-Natal (AE/Slotow/05).

Karongwe Game Reserve (KGR, center 24°13′S and 30°36′E), located in the Limpopo Province, South Africa, is an 85 km^2^ fenced private conservancy within the Granite Lowveld Bioregion of the Savanna Biome [21]. Mean annual rainfall is 515 mm (±70 SE), but animals have access to natural rivers and artificial waterholes throughout the year. For this study, we used a vegetation map of KGR that was categorized into five habitat types that differ in vegetation density: Closed riverine (1.6% of area), Open riverine (15.8%), Closed woodland (54.4%), Open woodland (24.1%), and Open scrub habitat (4.1%), in order of decreasing structural density [19].

We recorded group sizes of wildebeest and zebra throughout KGR during five sampling periods that spanned the rainy and dry seasons in 2004–2005, from 26–30 April 2004 (rainy; 78 mm rainfall during preceding month), 29 November – 3 December 2004 (rainy; 154 mm), 16–20 March 2005 (dry; 18.5 mm), 2–6 June 2005 (dry; 0 mm), and 1–5 September 2005 (dry; 0 mm). During each sampling period of five consecutive days, two teams travelled the reserve roads in opposite directions for six hours (0600–1100), and recorded the locations (lat/long coordinates) of all groups of target herbivore species. The starting and endpoints of each drive count were alternated daily to ameliorate researcher and time bias [22]. To avoid bias from interspecies interactions, we only considered single species groups in this study. Inter-individual distance within groups was typically less than six body lengths and groups were small (≤16 individuals) so group composition was unambiguous. We classified individuals as adult males, adult females and calves (young <1 year old of either sex).

During the study period, almost all lion (n = 4 of 5 in the reserve) were fitted with VHF transmitters (Telonics SB2 Transmitter, Africa Wildlife Tracking, Pretoria, South Africa) and were located daily by homing-in. The single uncollared lion was always located with a group of collared lions and thus complete information on the locations of all lion within the reserve was obtained. We used 2–4 daily point locations from all lions spread evenly over a 24 hr period to generate utilization distributions (UDs) of lion during the 30 days preceding each herbivore census. This 30-day period of lion locations sufficiently represents normal home-ranging activity [23] and was comparable to the seasonal patterns [24]. Utilization distributions were calculated from the pooled locations of all individuals using a 95% fixed kernel estimator with least-squares cross validation bandwidth selection [25] using Home Range Tools [26] in ArcGIS 9.3 (Environmental Systems Research Institute, Redlands, CA, USA). Thus, the UD values of lion constituted a robust measure of the probability of lion encounter at the landscape level [27].

**Figure 1:**
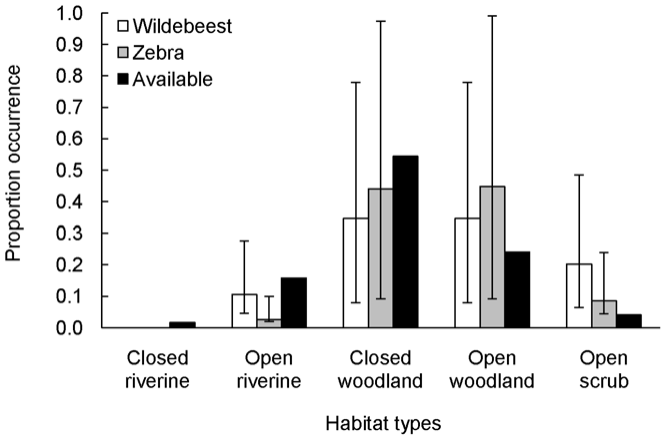
Occurrence of groups of wildebeest and zebra in the different habitats of Karongwe Game Reserve. Shown are the proportion occurrence of groups of wildebeest (n = 133, white bars) and zebra (n = 116, gray bars) with 95% confidence intervals. Proportions of available habitat (black bars) are listed in order of decreasing structural density.

At each location of a group, we used ArcGIS 9.3 to extract the UD values of lion and habitat type from the landscape map. We used χ^2^ goodness-of-fit tests to determine whether groups of wildebeest and zebra used habitats in proportion to availability. If a difference was found between use and available, we used Goodman's confidence intervals (graphically shown in Figure 1) to determine which habitats were selected or avoided [28]. We modeled group size for wildebeest and zebra as a function of habitat type, season and lion UD using generalized linear models in SPSS 15.0. For analyses, group size and lion UD values were square-root transformed and habitat type was included as dummy variables. After testing for multicollinearity, we excluded closed riverine and closed woodland habitats from analyses of wildebeest group size, and closed riverine and open woodland from analyses of zebra group size. All other predictor variables were included (tolerance levels >0.764 for all variables). Group composition, as measured by the proportion of females in each group (arcsin transformed for analysis), was similarly modeled using generalized linear models in SPSS 15.0. For these analyses, we excluded closed riverine and open woodland habitats for both species due to multicollinearity (tolerance levels >0.749 for all other variables).

We used an information-theoretic approach to test *a priori* models that best explain group size and the proportion of females in groups for each species on the landscape. Models contrasted the main effects of habitat type, season and lion UD, as well as the interactive effects of lion UD and open habitats (open woodland and open scrub). We used Akaike's Information Criterion corrected for small sample size (AIC_c_) to assess model weights (*w_i_*) and ranked candidate models using ΔAIC_c_ [29]. To account for model selection uncertainty, we averaged the estimates of the coefficients of main effect parameters in each model with ΔAIC_c_ ≤2 [29].

## Results

### Group size

Groups of wildebeest and zebra were found in all habitat types except closed riverine, but selection of habitats was not in proportion to availability (χ^2^ = 19.69, p<0.001 for wildebeest, χ^2^ = 20.23, p<0.001 for zebra; Figure 1). Groups of both species selected open scrub habitat more than expected based on availability, and zebra avoided open riverine (Figure 1). Closed woodland and open woodland habitats were selected in proportion to availability (Figure 1).

**Figure 2:**
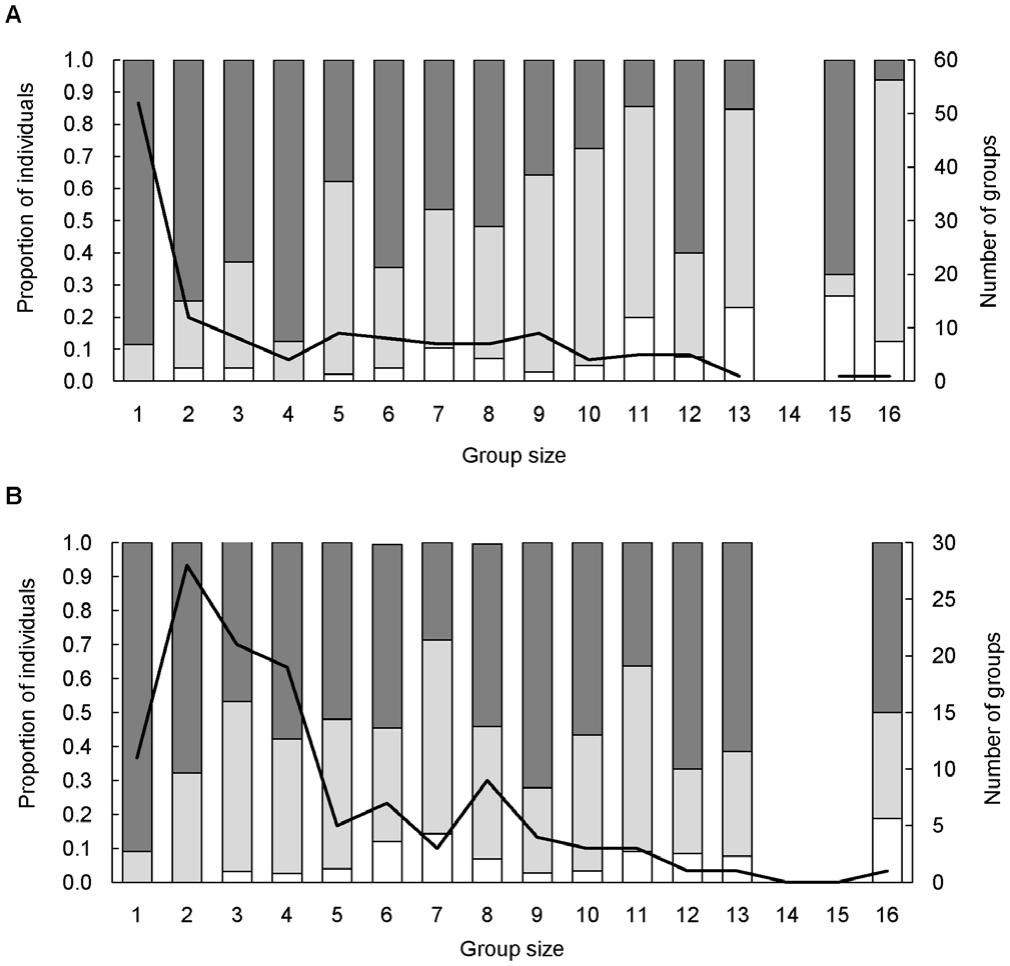
Species-specific group composition across the range of group sizes. Shown are the number of groups (line) and the proportion of adult males (dark bars),adult females (light bars), and young (white bars) for the range of group sizes of (A) wildebeest and (B) zebra.

**Figure 3:**
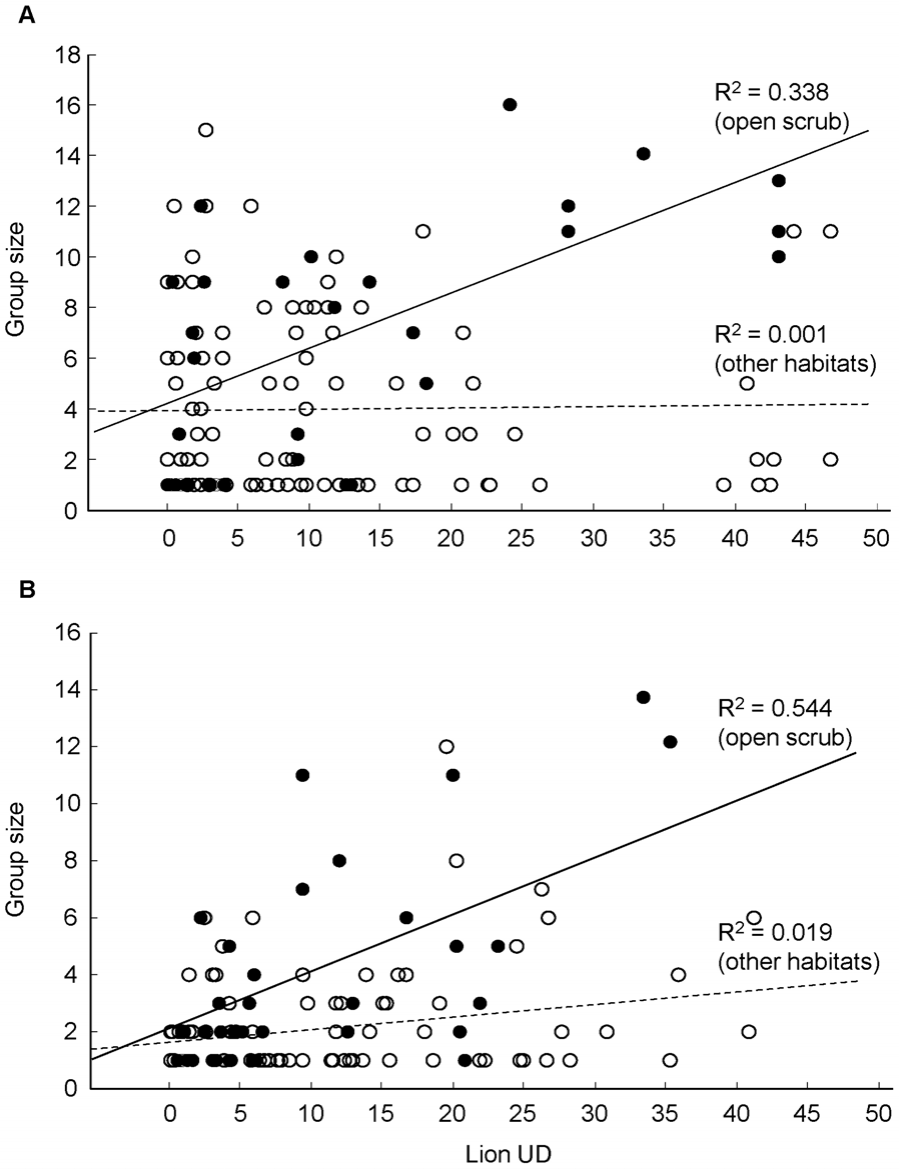
Habitat-specific relationship between group size and the probability of encountering lion. Shown are the regression lines for the relationship between the utilization distribution (UD) of lion and group size of (A) wildebeest and (B) zebra in open scrub (filled circles, solid line) and other habitats (open circles, dashed line).

Group sizes of wildebeest (n = 133) and zebra (n = 116) ranged from 1–16 individuals, and showed a typical right-skewed distribution where smaller groups were more common than larger groups (Figure 2). The size of wildebeest groups was best explained by a single model (*w_i_* = 0.69), while that for zebra was best explained by three top models (*w_i_* = 0.89; Table 1). Both wildebeest and zebra were found in larger groups in areas with high lion UDs (β = 0.018±0.005 SE for wildebeest; β = 0.109±0.079 SE for zebra), but were in smaller groups in open scrub habitat (β = −0.321±0.343 SE for wildebeest; β = −0.437±0.348 SE for zebra). There was, however, an interaction effect between these two parameters. In open scrub habitats, group size of wildebeest increased by 1.3 times (β = 0.259±0.100 SE, R^2^ = 0.338, Figure 3a) and those of zebra increased by 1.1 times (β = 0.101±0.084 SE, R^2^ = 0.544, Figure 3b) for every unit increase in lion UD. There was no relationship between lion UD and group size in other habitats (Figure 3).

**Table 1:** Best supported models that predict the group size and group composition of wildebeest (n = 133 groups) and zebra (n = 116 groups) in Karongwe Game Reserve.

Species	Predicting	Best supported models	*K*	AIC_c_	Δ AIC_c_	*w_i_*
Wildebeest	Group size	lion UD + open scrub + lion UD x open scrub	5	346.82	–	0.69
	Group composition	open riverine + closed woodland + open scrub	5	197.75	–	0.75
Zebra	Group size	lion UD + open scrub	4	238.18	–	0.49
		lion UD + open scrub + lion UD x open scrub	5	239.77	1.59	0.22
		lion UD	3	240.157	1.98	0.18
	Group composition	lion UD + season	4	113.57	0	0.38
		lion UD + open scrub + season	5	114.47	0.90	0.24
		season	3	114.84	1.27	0.20

Reported are the number of parameters (*K*, which includes the intercept β_0_ and residual variance σ^2^), Akaike's Information Criterion adjusted for small sample size (AIC_c_), distance from the lowest AIC_c_ (Δ AIC_c_), and Akaike's model weight (*w_i_*). Only models with Δ AIC_c_ <2 are shown for sake of clarity.

### Group composition

Groups of wildebeest and zebra in KGR comprised of mixed sexes and ages throughout the year (Figure 2). As group size increased, the proportion of females generally increased in wildebeest (Figure 2a), but remained relatively stable in zebra (Figure 2b). At the landscape level, the proportion of females in each group for these species was explained by parameters that differed from those that best explained group size (Table 1).

The composition of wildebeest groups was best explained by a single top model (*w_i_* = 0.75). Wildebeest groups had proportionally fewer females in open riverine (β = −0.388±0.151 SE), closed woodland (β = −0.085±0.102 SE) and open scrub (β = −0.024±0.118 SE) habitats. The composition of zebra groups was best explained by three top models (*w_i_* = 0.82). Zebra groups had proportionally fewer females in areas with high lion UDs (β = −0.040±0.032 SE) and in open scrub habitats (β = −0.043±0.048 SE). Zebra groups also comprised of higher proportions of females in the wet season compared to the dry season (β = 0.284±0.200 SE).

## Discussion

Gregarious ungulates display a quantitative as well as qualitative response to predation risk at the landscape level. The quantitative response of increasing group size with increasing risk of predation has been shown with considerable empirical evidence in a range of taxa [15], [30], [31]. The positive association between group size and probability of lion encounter (calculated as UDs) which we show, directly supports the results of Valeix et al. [32] for these two species in the larger Hwange National Park, Zimbabwe. Individuals in larger groups are predicted to experience lower predation risk because probability of capture decreases [33], [but see 34], despite the possible increase in the probability of attack [35]. For zebra and wildebeest, larger groups are safer in the open plains of the Serengeti as lion prefer attacking smaller groups [10].

In KGR, lion hunt zebra and wildebeest in a mixed mosaic of riverine, woodland and open scrub habitats [24]. Thus, the relationship between predation risk and group size for these ungulates is expected to depend on habitat type. Despite the low availability of open scrub in this landscape, both species selected this habitat type. Furthermore, group sizes in open scrub were affected by predation risk, while those in all other habitats were not. In fact, group sizes remained lower in other habitats even though the probability of lion encounter was as high as that in open scrub. Formation of larger groups in open habitats has been recorded in other gregarious ungulates such as bison, *Bison bison*, and elk, *Cervus elaphus*. For these species, grouping in open habitats is more a function of resource requirements than a response to higher perceived predation risk from wolves, *Canis lupus* [2], [ but see 8], [36]. This may be because the hunting patterns and activity areas of coursing hunters, such as wolf, are typically spatially divergent [36], while stalk-and-ambush hunters, such as lion, opportunistically utilize ambush sites within their territory [37]. Thus for African ungulates, forming larger groups in open habitats within lion activity areas can be an effective antipredator strategy, especially since the capture probability of lion decreases when they attack larger groups that are farther from cover [38].

Factors explaining the composition of groups for these ungulate species differed from those explaining group sizes. This was expected given the seasonal and sex differences in predation risk for these species. In Kruger National Park, South Africa, predation risk from lion was highest for male wildebeest during the early dry season but for females, it was highest in the early wet season [12]. Despite differences between the sexes in vulnerability across seasons, we found no difference in group composition as a function of lion activity or season in wildebeest. By contrast, zebra groups in KGR comprised of more females during the rainy season, which corresponded to the calving period, a time when vulnerability to predation risk is highest. Females of zebra are also more vulnerable than males to predation by lions in general [12], [20]. Thus, it was not surprising that groups of zebra with higher proportions of females were more risk averse, by avoiding areas with high lion activity and avoiding open scrub habitats where they would be more conspicuous. Wildebeest groups with proportionally more females also avoided certain habitat types, but this response was likely a combination of both risk aversion (open scrub) and resource selection (open riverine and closed woodland).

In KGR, zebra and wildebeest have similar population densities and relative predation impact from lion [19]. These species also typically have similar spatial distributions centered on open savanna, suggesting similar broad resource requirements and tolerances [39]. Thus, differences between zebra and wildebeest in group composition were unlikely to be due to population densities, overall predation risk, or broad resource selection. However, differences in the social strategies between these species may explain the changes in group composition as group size increases (Figure 2). Zebra occur in stable family groups, comprising of one dominant male with several females and their associated young [40]. When families of zebra merge, thereby increasing group size, the resulting proportion of females to males continues to remain stable (Figure 2b). By contrast, groups of wildebeest females and their young utilize areas within the territories of males [40]. Thus when groups of wildebeest merge, they are centered within the territories of fewer males, thereby skewing the sex ratio towards females (Figure 2a). Differences in gut anatomy and therefore digestive tolerance, between these two grazers can also lead to differences in the composition of groups and the associated costs of habitat selection across the landscape. Zebra, a non-ruminant grazer, can tolerate a wider range of grass quality than wildebeest, but must obtain a higher daily food intake to meet their metabolic requirements [41]. This difference in physiological constraints may explain why females of wildebeest, but not zebra, avoided open riverine and closed woodland habitats, where grass quality is poor. Furthermore, the cost of avoiding high resource open scrub habitats by females is likely to differ between these two species. Females of zebra miss opportunities to graze frequently [42], while females of wildebeest miss opportunities to access high quality short grasses [41]. Such losses of foraging opportunities, irrespective of the probability of encountering lion, are likely to be particularly costly in areas such as KGR, where open scrub habitat is limited.

In summary, theoretical [43] and empirical evidence from a range of taxa predict that grouping is favored in open habitats and when predation risk is high. Our results with zebra and wildebeest in KGR directly support this pattern in a predominantly woodland landscape. The long-term activity areas of stalk-and-ambush predators such as lion are predictably dangerous for prey, and we show that this measure of predation risk can affect the group sizes of two of their major prey. The predator-induced quantitative response (increase in group size) was only apparent in open scrub habitats, reinforcing the observation that woodlands appear safer than open scrub habitats for ungulates in general [2], [8], [44], [45]. Group composition however, seemed to reflect specific social, physiological and foraging constraints of the two species, as well as predation risk. Despite the larger group sizes in open scrub habitat, females of both species avoided this high resource area, resulting in a potential foraging cost associated with minimizing predation risk. Thus, landscape-level grouping dynamics are species specific and particular to the composition of the group, arising from a tradeoff between maximizing resource selection and minimizing predation risk.
